# Inhibition of PKA/CREB1 pathway confers sensitivity to ferroptosis in non-small cell lung cancer

**DOI:** 10.1186/s12931-023-02567-3

**Published:** 2023-11-13

**Authors:** Guangyao Shan, Guoshu Bi, Guangyin Zhao, Jiaqi Liang, Yunyi Bian, Huan Zhang, Xing Jin, Zhengyang Hu, Guangyu Yao, Hong Fan, Cheng Zhan

**Affiliations:** 1 Department of Thoracic Surgery, Zhongshan Hospital, Fudan University, Shanghai, China; 2 Division of Thoracic Surgery, School of Medicine, Sichuan Cancer Hospital and Research Institute, University of Electronic Science and Technology of China (UESTC), Chengdu, China

**Keywords:** Ferroptosis, Lipid metabolism, Transcription factor, Non-small cell lung cancer

## Abstract

Ferroptosis is a type of regulated cell death characterized by iron accumulation and lipid peroxidation. The molecular mechanisms underlying ferroptosis regulation in non-small cell lung cancer (NSCLC) are poorly understood. In this study, we found that protein kinase A (PKA) inhibition enhanced ferroptosis susceptibility in NSCLC cells, as evidenced by reduced cell viability and increased lipid peroxidation. We further identified cAMP-responsive element protein 1 (CREB1), a transcription factor and a substrate of PKA, as a key regulator of ferroptosis. Knockdown of CREB1 sensitized NSCLC cells to ferroptosis inducers (FINs) and abolished the effects of PKA inhibitor and agonist, revealing the pivotal role of CREB1 in ferroptosis regulation. Using a high-throughput screening approach and subsequent validation by chromatin immunoprecipitation (ChIP) and dual-luciferase assays, we discovered that CREB1 transcriptionally activated stearoyl-CoA desaturase (SCD), an enzyme that catalyzes the conversion of saturated fatty acids to monounsaturated fatty acids. SCD conferred ferroptosis resistance by decreasing the availability of polyunsaturated fatty acids for lipid peroxidation, and its overexpression rescued the effect of CREB1 knockdown on ferroptosis in vitro. Besides, CREB1 knockdown suppressed xenograft tumor growth in the presence of Imidazole Ketone Erastin (IKE), a potent FIN, and this effect was reversed by SCD. Finally, we showed that high expression of CREB1 was associated with poor prognosis in NSCLC patients from public datasets and our institution. Collectively, this study illustrates the effect of PKA/CREB1/SCD axis in regulating ferroptosis of NSCLC, targeting this pathway may provide new strategies for treating NSCLC patients.

**Supplementary Information:**

The online version contains supplementary material available at 10.1186/s12931-023-02567-3.

## Introduction

Epidemiological data has revealed that lung cancer is one of the most prevalent types of malignant neoplasm and the leading cause of cancer-related death globally, in which non-small cell lung cancer (NSCLC) accounts for 85% of cases [1, 2]. Despite advances in diagnosis and treatment, the prognosis of NSCLC remains dismal, highlighting the need for novel therapeutic strategies [3].

Ferroptosis, unlike apoptosis and autophagy, is distinguished by the accumulation of reactive oxygen species (ROS) and the subsequent lipid peroxidation catalyzed by iron [4]. It has been frequently reported that dedifferentiated cancer cells which have been resistant to apoptosis and conventional therapies are still vulnerable to ferroptosis inducers (FINs) [5], underlining the role of ferroptosis in cancer management. Thus, elucidating the underlying mechanisms of ferroptosis is warranted.

Up to now, substantial evidence has uncovered the critical role of lipid metabolism in ferroptosis [6, 7]. The execution of ferroptosis depends on polyunsaturated fatty acids (PUFAs), which act as the fuel for lipid peroxidation, while monounsaturated fatty acids (MUFAs) could compete with PUFAs for integration into membrane phospholipids, thus reducing the availability of oxidizable PUFAs to protect cells from ferroptosis [8, 9]. Stearoyl-CoA desaturase (SCD) is an enzyme responsible for the conversion of saturated fatty acids (SFAs) to MUFAs and hence plays a critical anti-ferroptosis role in carcinogenesis and treatment of malignancies [10–12].

As a well-characterized substrate of protein kinase A (PKA) and a transcription factor, the expression and phosphorylation of cAMP-responsive element protein 1 (CREB1) are upregulated in tumor tissue compared to adjacent normal tissue in NSCLC [13]. What’s more, the overexpression of CREB1 and phospho-CREB1 is negatively associated with survival in NSCLC patients [14]. Previous studies revealed that PKA promotes cell migration, epithelial-mesenchymal transformation, and sensitizes lung cancer cells to radiotherapy [13]. However, the effect of PKA/CREB1 pathway on ferroptotic cell death remains to be fully elucidated.

The purpose of this study was to clarify the role of the PKA/CREB1 pathway in regulating NSCLC ferroptosis and investigate the underlying mechanisms using cell viability assays, lipid peroxidation detection, and pseudotargeted lipidomic analysis. Targeting PKA/CREB1 pathway may enhance ferroptosis and provide a new therapeutic option for NSCLC patients.

## Methods

### Cell lines and agents

Human NSCLC cell lines A549, H1299, and human embryonic kidney cell HEK293T were obtained from the Chinese Academy of Sciences cell bank and authenticated by Short Tandem Repeat (STR) in September 2022. DMEM high glucose medium (KeyGEN BioTECH, Jiangsu Province, China) containing 10% fetal bovine serum (EVERY GREEN, TIANHANG Biotechnology, Zhejiang Province, China), 0.1 mg/ml streptomycin, 100 U/ml penicillin, and 0.25 μg /ml amphotericin B (Beyotime, Shanghai, China) was used for cell culture. The cells were maintained in a humidified atmosphere with 95% air and 5% CO_2_. Reagents including FINs RSL3 and Imidazole Ketone Erastin (IKE), ferroptosis inhibitors ferrostatin-1 (ferr-1) and defetoxamine (DFO), necroptosis inhibitor necrosulfonamide (necro), apoptosis inhibitor Z-VAD (OMe)-FMK (Z-VAD-FMK), PKA inhibitor H-89 dihydrochloride (H89), and PKA activator 8-Bromo-cAMP sodium salt (cAMP) were provided by TargetMol (Boston, MA, USA) and dissolved in dimethyl sulfoxide (DMSO).

### Transfection of siRNA, shRNA, and overexpression lentivirus

Three siRNAs targeting CREB1 were purchased from RIBOBIO Corporation (Guangdong Province, China), and transfected into cells in the presence of lipo8000 (Beyotime) and Opti-MEM medium (Gbico, Carlsbad, CA, USA) according to the manufacturer’s protocols. The sequences of siCREB1-1 (5’-GCTCGAGAGTGTCGTAGAA-3’) and siCREB1-2 (5’-GAGTCAGTGGATAGTGTAA-3’) were cloned into puromycin-resistant lentivirus vector (Genechem, Shanghai, China) and screened in the presence of puromycin for 48 h. Similarly, the SCD overexpression lentivirus (Genechem) was constructed and transfected.

### qRT-PCR

The total RNA extraction was conducted using TRIzol reagent (TIANGEN, Beijing, China) following the manufacturer's protocols. Subsequently, qRT-PCR was performed in an ABI QuantStudio 5 real-time PCR system (Thermo Fisher Scientific, Waltham, MA, USA) by using Hifair II First-Strand cDNA Synthesis Kit (YEASEN, Shanghai, China) and Hieff qPCR SYBR Green Master Mix (YEASEN). The samples were added in triplicates and the relative mRNA expression was determined using the 2^−ΔΔCT^ method. β-actin was used as an endogenous control. All the primers were synthesized by Sangon Biotech (Shanghai, China) and were listed in Additional file 1.

### Immunoblotting

Cells were lysed using RIPA buffer (Beyotime) mixed with a protease inhibitor (TargetMol) and two phosphatase inhibitor cocktails (TargetMol) for 10 min on ice. The protein concentrations were quantified using a BCA assay (YEASEN). Proteins were separated by Sodium Dodecyl Sulphate–Polyacrylamide Gel Electrophoresis (YAMAY Biotech, Shanghai, China) and then they were transferred to the polyvinylidene fluoride (PVDF) membranes (Millipore, Billerica, MA, USA). Subsequently, the membranes were blocked with 5% non-fat milk at room temperature for 1 h, and then they were incubated with corresponding primary antibodies overnight at 4 °C. After being washed three times with Tris-buffered saline-Tween (TBST) solution, the membranes were incubated with horseradish peroxidase (HRP)-labeled secondary antibodies (1:2,500, Beyotime) for 1 h at room temperature. BeyoECL Moon Chemiluminescence Reagent (Beyotime) was used for protein detection. The information of primary antibodies was provided in Additional file 1.

### Cell viability assays

For cytotoxicity assays, the cells were seeded at a density of 5,000 cells per well in quadruplicate in 96-well plates and maintained in a cell incubator for 24 h. Cells were administered with gradients of RSL3/IKE with or without pre-treatment of H89/cAMP for 48 h. Cell viability was measured with Cell Counting kit-8 (TargetMol) according to the manufacturer’s instructions and the optical density (OD) value of each well was determined on a microplate reader.

### Detection of lipid peroxidation

After pretreatment with corresponding reagents, the cells were harvested and washed with PBS. Subsequently, the cells were resuspended in DMEM medium containing 200 μM BODIPY 581/591 C11 dye (Thermo Fisher Scientific) for lipid-ROS detection and then were maintained in a cell incubator for 30 min. At last, the cells were washed with PBS two times, and the level of lipid-ROS was determined with an Accuri 6 cytometer (BD Biosciences, San Jose, CA, USA) through FITC channel. The results generated by flow cytometry were visualized using FlowJo software (TreeStar Inc, Woodburn, OR, USA).

### Measurement of the malondialdehyde (MDA) level

Cells were planted in 6-well plates and were lysed using RIPA buffer (Beyotime) at a confluence of 80% to 90%. After centrifugation at 12,000 rpm for 15 min, the supernatant was extracted for further analysis. The protein quantification was performed by using a BCA assay (YEASEN), and the MDA concentration was colorimetrically determined based on the reaction with thiobarbituric acid following the instructions of Lipid Peroxidation MDA Assay Kit (Beyotime). The MDA concentration of the total protein per microgram was used to compare the MDA production of each group.

### Pseudotargeted lipidomics analysis

1 × 10^6^ A549 cells transfected with NC or CREB1-knockdown lentivirus were seeded in 6 cm dishes and treated with RSL3 (2 μM) for 2 h on the next day. After treatment, cells were washed with PBS, harvested using a cell scraper, and then centrifuged. Liquid nitrogen was used to freeze the cell pellets before they underwent a modified Folch lipid extraction process [15]. In a nutshell, the A549 cells were resuspended using 600 μL methyl alcohol/water (1:1, v/v) solution containing isotope-labeled internal mix standards provided by Avanti Polar Lipids (Birmingham, AL, USA) and Sigma-Aldrich supplied (St. Louis, MO, USA). Subsequently, 600 μL chloroform was added, and the samples were subjected to ultrasonication for 3 min and ultrasonic extraction in an ice-water bath for 10 min, followed by incubation at 4℃ for 30 min, and the chloroform layers were collected and lyophilized in a centrifugal vacuum evaporator. The identical conditions as aforementioned were used to extract the residue once again. In the end, samples were mixed and reconstituted in isopropanol/methanol (1:1, v/v), vortexed for 30 s, sonicated for 3 min, and centrifuged at 13,000 rpm for 10 min at 4 °C. 200 μL supernatant of each sample was collected for further analysis. The quality-control sample was made by combining aliquots of the supernatants from each sample in equal volume. The Liquid chromatography with tandem mass spectrometry (LC–MS) analysis was conducted by Lumin Biological Technology, LTD (Shanghai, China).

### Chromatin immunoprecipitation (ChIP) assay

The SimpleChIP Plus Enzymatic Chromatin IP Kit (Cell Signaling Technology, Danvers, MA, USA) was used for the assay following the manufacturer's instructions. Firstly, cells were fixed with formaldehyde to cross-link proteins and DNA. Then, chromatin was cut down into 150–900 bp DNA/protein fragments by Micrococcal Nuclease. Next, IgG or an anti-CREB1 antibody (1:50, #9197, Cell Signaling Technology) was added. After the complex was co-precipitated, Protein G magnetic beads were exploited to capture the complex. The cross-links were then reversed after the chromatin was eluted. Finally, DNA fragments were purified using spin columns and quantified by qRT-PCR assay. The information of primers used for ChIP-qPCR was listed in Additional file 1.

### Dual-luciferase reporter assay

The promoter region of SCD spanning from -2000 to + 200 and the corresponding mutated sequence was cloned into the phy-811 dual luciferase reporter vector (Hanyin Technology, Shanghai, China). The NC and CREB1-knockdown HEK-293 T cells were co-transfected with SCD-WT or Mut plasmids and renilla luciferase reporter plasmids using Lipo8000 (Beyotime) at a confluence of 60–80%. Cells were harvested at 48 h after transfection. Next, the dual-luciferase reporter assay was performed using a Luciferase Reporter Gene Assay Kit (Beyotime) following the manufacturer’s instructions, and luciferase activity was detected using FlexStation 3 Microplate Reader (Molecular Devices, San Jose, CA, USA).

### Animal experiments

The animal experiments were conducted in compliance with the guidelines of the animal ethics committee of Zhongshan Hospital, Fudan University. The nude mice were obtained from Shanghai Jiesijie Laboratory Animal Company and maintained in laminar flow cabinets under specific pathogen-free conditions. A549 cells with different treatments (NC + vector, CREB1-SH1 + vector, NC + SCD-OE, CREB1-SH1 + SCD-OE) were suspended in 100 μL PBS and subcutaneously inoculated into the right flank of each mouse (2 × 10^6^ cells per mouse). When the tumor volume reached about 50 mm^3^, the mice were randomly assigned to the DMSO or IKE (30 mg/kg) group and then intraperitoneally injected every three days for six times. The body weight of the mice was not significantly affected by the treatment. The tumor size was measured weekly using vernier calipers and the tumor volume was calculated as (length × width^2^) / 2. After four weeks of implantation, the mice were euthanized and the tumors were harvested for immunohistochemistry (IHC) analysis.

### Patient selection

We conducted a retrospective analysis of 120 patients with lung adenocarcinoma (LUAD) and 78 patients with lung squamous cell carcinoma (LUSC) who underwent surgical resection at the Department of Thoracic Surgery, Zhongshan Hospital, Fudan University from 2016 to 2019 (Additional file 2). The surgical procedures were performed by experienced thoracic surgeons and the histopathological diagnosis of the tumors and lymph nodes was confirmed by at least two qualified pathologists. The tumor stage was assigned according to the eighth edition of the International Union Against Cancer (UICC) Cancer Staging Manual using the tumor/lymph node/metastasis (TNM) classification. The follow-up was terminated in June 2021. Overall survival (OS) was defined as the time from surgery to death or the last follow-up for censored patients. Recurrence-free survival (RFS) was defined as the time from surgery to tumor recurrence. This study was conducted in accordance with the Declaration of Helsinki and approved by the Research Ethics Committee of Zhongshan Hospital, Fudan University (approval number: B2022-180R).

### IHC

The formalin-fixed and paraffin-embedded xenograft tumor tissues from executed mice and resected tumor tissues from NSCLC patients were prepared as slices. Next, they were dewaxed, rehydrated, and stained using the GTVisionTM + Detection System/Mo&Rb Immunohistochemistry kit following the instructions of the manufacturer (GeneTech, Shanghai, China). The antibodies used for IHC staining were as follows: Anti-4 Hydroxynonenal (4-HNE) antibody (1:50, ab48506, Abcam, Cambridge, MA, USA), anti-phospho-CREB1 (Ser133) antibody (1:100, CY5043, Abways, Shanghai, China), anti-CREB1 antibody (1:100, CY5426, Abways), and anti-SCD antibody (1:100, abs14771, Absin, Shanghai, China).

### RNA sequencing and bioinformatics analysis

The total RNA of control and CREB1-knockdown A549 cells was extracted using TRIzol reagent (TIANGEN) and sequenced by OE Technology (Shanghai, China). The raw data was normalized to Fragments Per Kilobase of exon model per Million mapped fragments (FPKM) format for subsequent analysis.

We used the cor.test function and the ggplot2 package in R software (Version 4.1.1, R Foundation for Statistical Computing, Vienna, Austria) to calculate and visualize the Pearson correlation between the expression of CREB1 and the corresponding biological pathways. The pathway enrichment analyses of CREB1 in LUAD and LUSC tissue were retrieved from CAMOIP (http://www.camoip.net/). The Pearson correlation between CREB1 and SCD expression in LUAD and LUSC tissue was obtained from GEPIA2 (http://gepia2.cancer-pku.cn/). The expression difference of CREB1 and prognostic analysis from public datasets were generated by UALCAN (https://ualcan.path.uab.edu/) and KM plotter (https://kmplot.com/), respectively.

### Statistical analyses

Statistical analyses were carried out by using R software and GraphPad Prism 9 (GraphPad Software, La Jolla, CA, USA). Student’s t‐test or Wilcoxon test was exploited for comparison of continuous variables when appropriate. The survival analysis was performed by Kaplan–Meier survival analysis with the log-rank test. All the tests were two-sided and the threshold of p-value for statistical significance was set as 0.05.

## Results

### The PKA/CREB1 pathway is activated during ferroptosis

**Fig. 1:**
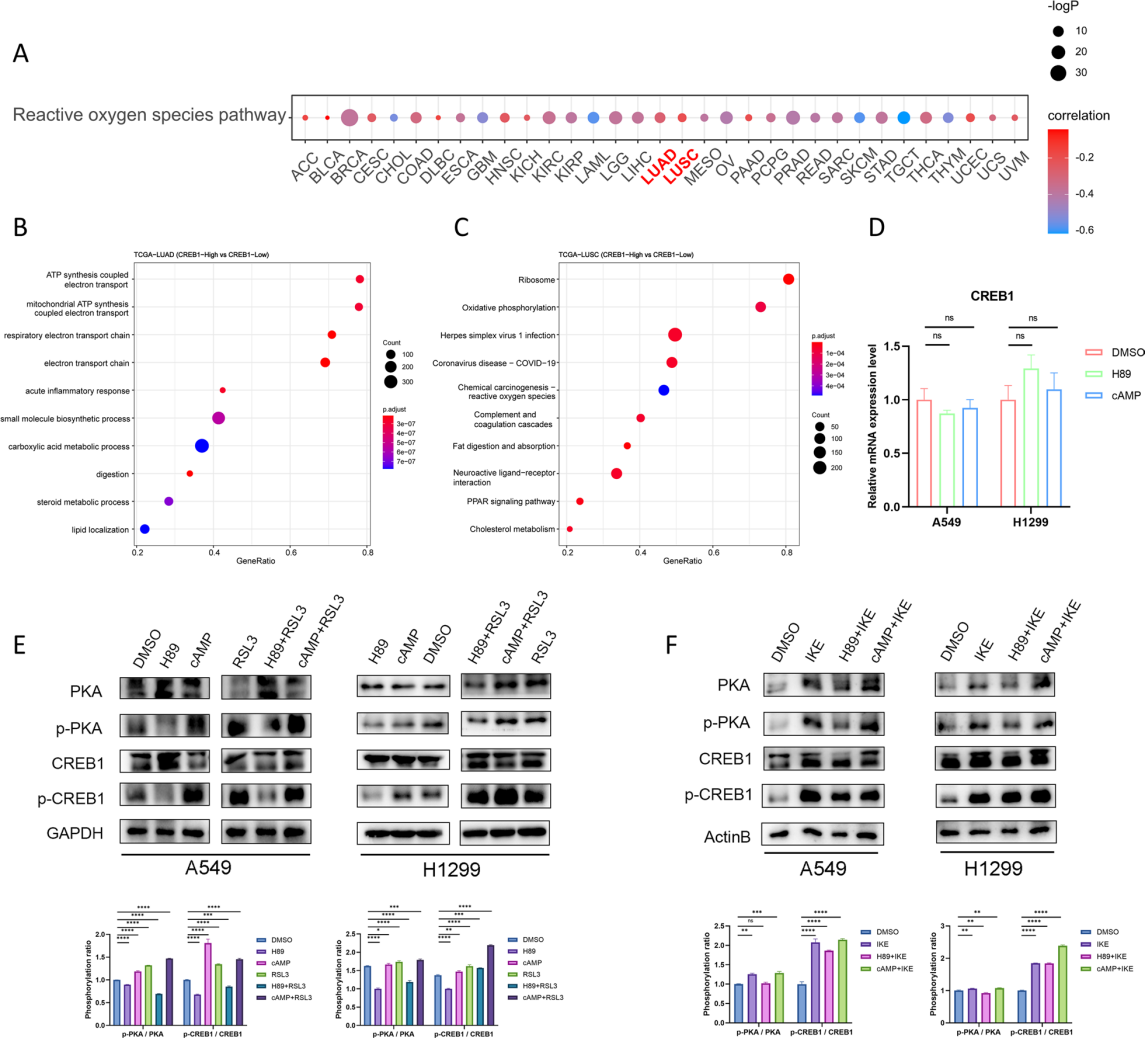
PKA/CREB1 pathway is activated during ferroptosis. **A** Pan-cancer analysis of the Pearson correlation between the expression of CREB1 and activity of reactive oxygen species pathway. **B**, **C** Enrichment analysis demonstrated the top 10 enriched biological pathways between TCGA-LUAD (B) and LUSC (C) patients with high or low expression of CREB1. **D** The relative mRNA expression of CREB1 in A549 and H1299 cells when treated with DMSO, H89 (PKA inhibitor, 30 μM), or cAMP (PKA activator, 100 μM) for 48 h. **E**, **F** The protein level and corresponding quantification of PKA, phospho-PKA, CREB1, and phospho-CREB1 in A549 and H1299 cells (cropped blots) treated with DMSO, H89 (30 μM), cAMP (100 μM), RSL3 (ferroptosis inducer, 0.2 μM for A549, 0.05 μM for H1299), IKE (ferroptosis inducer, 2 μM), or their combinations for 48 h. TCGA, the Cancer Genome Atlas; LUAD, lung adenocarcinoma; LUSC, lung squamous cell carcinoma; H89, H-89 dihydrochloride; cAMP, 8-Bromo-cAMP sodium salt; IKE, Imidazole Ketone Erastin. Data were analyzed by Student’s t-test and presented by mean ± SD in triplicate. ns, not significant; *, p < 0.05; **, p < 0.01; ***, p < 0.001; ****, p < 0.0001

We examined the relationship between CREB1 expression and the ROS pathway activity across different cancer types (Fig. 1A). The result demonstrated that CREB1 expression was inversely correlated with the ROS pathway activity. We then performed pathway enrichment analyses of the differentially expressed genes between high and low CREB1 expression groups in LUAD and LUSC patients. We observed that the genes were significantly enriched in ATP synthesis coupled electron transport and respiratory electron transport chain pathway (Fig. 1B, C). These pathways are involved in the generation of ROS, which can cause mitochondrial membrane potential hyperpolarization, lipid peroxide accumulation, and ferroptosis [16]. Therefore, we hypothesized that CREB1 may have an anti-ferroptotic function in NSCLC. Through qPCR and immunoblotting assays, we found the PKA agonist cAMP or inhibitor H89 didn’t have a significant impact on the mRNA expression of CREB1, but they modulated the phosphorylation levels of PKA (Thr197) and CREB1 (Ser133) (Fig. 1D, E). We also treated NSCLC cells with RSL3 (0.2 μM for A549, 0.05 μM for H1299) or IKE (2 μM) for 48 h, which are potent FINs. The phosphorylation levels of PKA and CREB1 were elevated in response to RSL3 or IKE (Fig. 1E, F), suggesting that the PKA/CREB1 pathway was activated during ferroptosis.

### H89/cAMP confers NSCLC cells more suspectable or resistant to ferroptosis

**Fig. 2:**
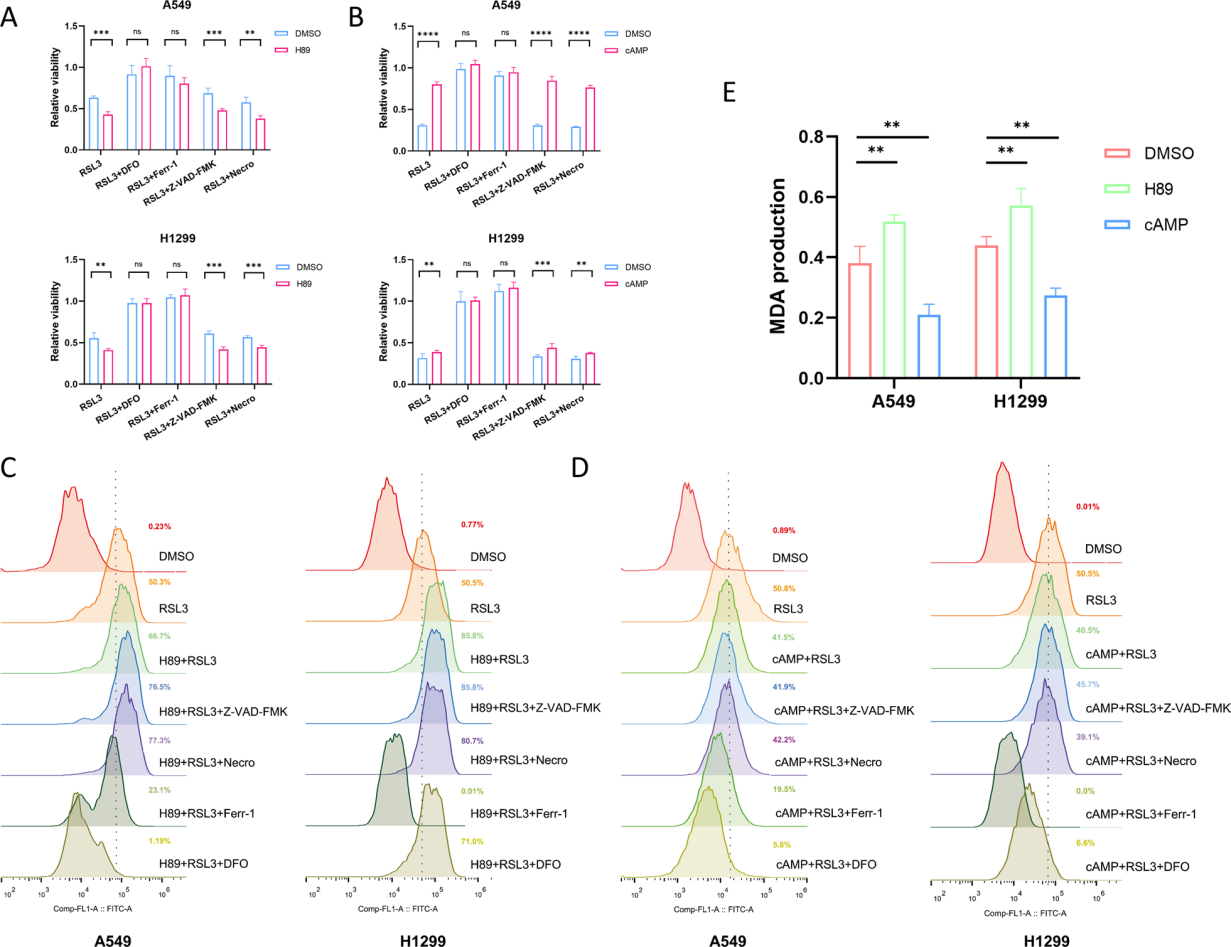
H89/cAMP confers susceptibility or resistance to ferroptosis. **A**, **B** Relative viability of A549 and H1299 cells treated with RSL3 (ferroptosis inducer, 2 μM), RSL3 (2 μM) plus DFO (ferroptosis inhibitor, 100 μM), RSL3 (2 μM) plus ferr-1 (ferroptosis inhibitor, 10 μM), RSL3 (2 μM) plus Z-VAD-FMK (apoptosis inhibitor, 10 μM), or RSL3 (2 μM) plus Necro (necroptosis inhibitor, 0.5 μM) after incubating with DMSO, H89 (30 μM) or cAMP (100 μM) for 48 h. **C**, **D** The lipid peroxidation level of A549 and H1299 cells detected by BODIPY-C11 dye while treated with RSL3 (2 μM), RSL3 (2 μM) plus DFO (100 μM), RSL3 (2 μM) plus ferr-1 (10 μM), RSL3 (2 μM) plus Z-VAD-FMK (10 μM), or RSL3 (2 μM) plus Necro (0.5 μM) after incubating with H89 (30 μM) or cAMP (100 μM) for 48 h. **E** Measurement of MDA production of A549 and H1299 cells incubating with RSL3 (2 μM) for 8 h after pre-treatment with DMSO, H89 (30 μM), or cAMP (100 μM) for 48 h. DFO, deferoxamine; ferr-1,ferrostatin-1; Z-VAD-FMK, Z-VAD (OMe)-FMK; Necro, Necrosulfonamide; MDA, malondialdehyde. The data are presented as the mean ± SD from three biological replicates. ns, not significant; **, p < 0.01; ***, p < 0.001; ****, p < 0.0001

We performed cell viability assays to evaluate the effect of PKA/CREB1 pathway on ferroptosis in NSCLC cells. We found that pre-treatment with H89 (30 μM), a PKA inhibitor, sensitized A549 and H1299 cells to RSL3 (2 μM), and this effect was reversed by ferr-1 (20 μM) or DFO (100 μM), which are ferroptosis inhibitors, but not by Z-VAD-FMK (10 μM, apoptosis inhibitor) or necro (1 μM, necroptosis inhibitor) (Fig. 2A). Conversely, pre-treatment with cAMP (100 μM), a PKA agonist, protected A549 and H1299 cells from RSL3-induced ferroptosis (Fig. 2B).

Subsequently, we measured the lipid peroxidation levels in different groups. We observed that H89 increased (Fig. 2C), while cAMP decreased (Fig. 2D) the lipid peroxidation levels in NSCLC cells. These effects were not affected by Z-VAD-FMK or Necro. Lipid peroxidation can cause membrane damage and generate toxic aldehydes such as MDA or 4-HNE, which can modulate various cellular processes and enhance ferroptosis by crosslinking and inactivating proteins [17]. Therefore, we assessed the levels of MDA, a product of lipid peroxidation, and confirmed the pro-ferroptotic role of H89 and the anti-ferroptotic effect of cAMP (Fig. 2E) in NSCLC cells. These results suggest that the inhibition of PKA/CREB1 pathway increases the susceptibility of NSCLC cells to ferroptosis.

### CREB1 is essential in regulating ferroptosis

**Fig. 3:**
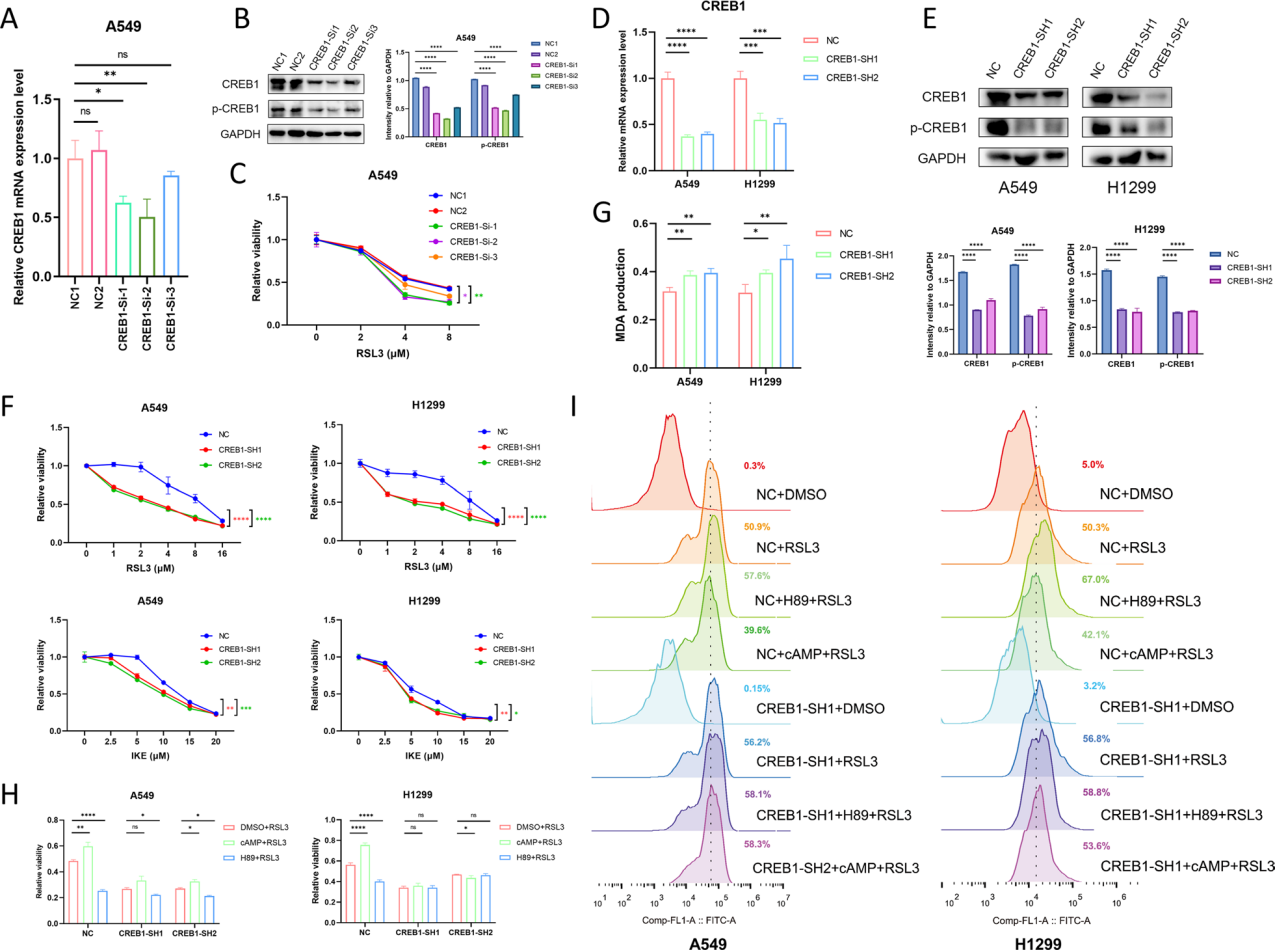
CREB1 plays a cardinal role in suppressing ferroptosis. **A**, **B** RT-qPCR (A) and immunoblotting (B, cropped blots) assays revealed the knockdown effect of three siRNAs targeting CREB1 in A549 cells. **C** Relative cell viability of A549 cells transfected with siNC (control siRNA) or siCREB1 treated with RSL3 in gradient concentrations. **D**, **E** RT-qPCR (D) and immunoblotting (E, cropped blots) assays revealed CREB1 mRNA expression in A549 and H1299 cells after transfection with NC (control shRNA), CREB1-SH1, or CREB1-SH2 lentivirus. **F** Relative cell viability of A549 and H1299 cells treated with RSL3 in gradient concentrations after transfection with NC, CREB1-SH1, or CREB1-SH2 lentivirus. **G** MDA production of A549 and H1299 cells after treatment with RSL3 (2 μM) for 8 h. **H** Relative cell viability of A549 and H1299 cells treated with DMSO plus RSL3 (2 μM), H89 (30 μM) plus RSL3 (2 μM), or cAMP (100 μM) plus RSL3 (2 μM) after transfection with NC, CREB1-SH1, or CREB1-SH2 lentivirus. **I** Lipid peroxidation measurement of different groups with or without pre-treatment of H89 (30 μM) or cAMP (100 μM) for 48 h. Data were analyzed by two-way ANOVA or Student’s t-test and presented by mean ± SD in triplicate. ns, not significant; *, p < 0.05; **, p < 0.01; ***, p < 0.001; ****, p < 0.0001

As a well-characterized substrate of PKA, we further explored the effect of CREB1 on ferroptosis. We constructed a CREB1-knockdown cell model by siRNA transfection and verified the silencing efficiency by qRT-PCR (Fig. 3A) and immunoblotting (Fig. 3B) assays. The cell toxicity assay suggested that the downregulation of CREB1 rendered A549 cells exquisitely sensitive to RSL3 (Fig. 3C). We then cloned the sequences of siCREB1-1 and siCREB1-2 into puromycin-resistant lentivirus vectors and confirmed the knockdown effect by qRT-PCR (Fig. 3D) and immunoblotting (Fig. 3E) assays in NSCLC cells. The knockdown of CREB1 sensitized A549 and H1299 cells to RSL3 and IKE, both of which are ferroptosis inducers (Fig. 3F). Subsequently, we assessed the levels of MDA, a marker of ferroptosis, and confirmed the anti-ferroptotic role of CREB1 (Fig. 3G).

We also pre-treated NSCLC cells with H89 (30 μM) or cAMP (100 μM) for 48 h and evaluated the effect of PKA inhibition or activation on ferroptosis. We noticed that the effect of H89 and cAMP was diminished in CREB1-knockdown cells (Fig. 3H), suggesting that CREB1 is essential for PKA-mediated ferroptosis regulation. Subsequently, We measured the lipid peroxidation levels by BODIPY-C11 and found that CREB1 knockdown increased lipid peroxidation and reduced the influence of H89 and cAMP on ferroptosis (Fig. 3I). These results indicate that CREB1 plays a cardinal role in inhibiting ferroptosis in NSCLC cells.

### CREB1 promotes SCD expression to inhibit ferroptosis

**Fig. 4:**
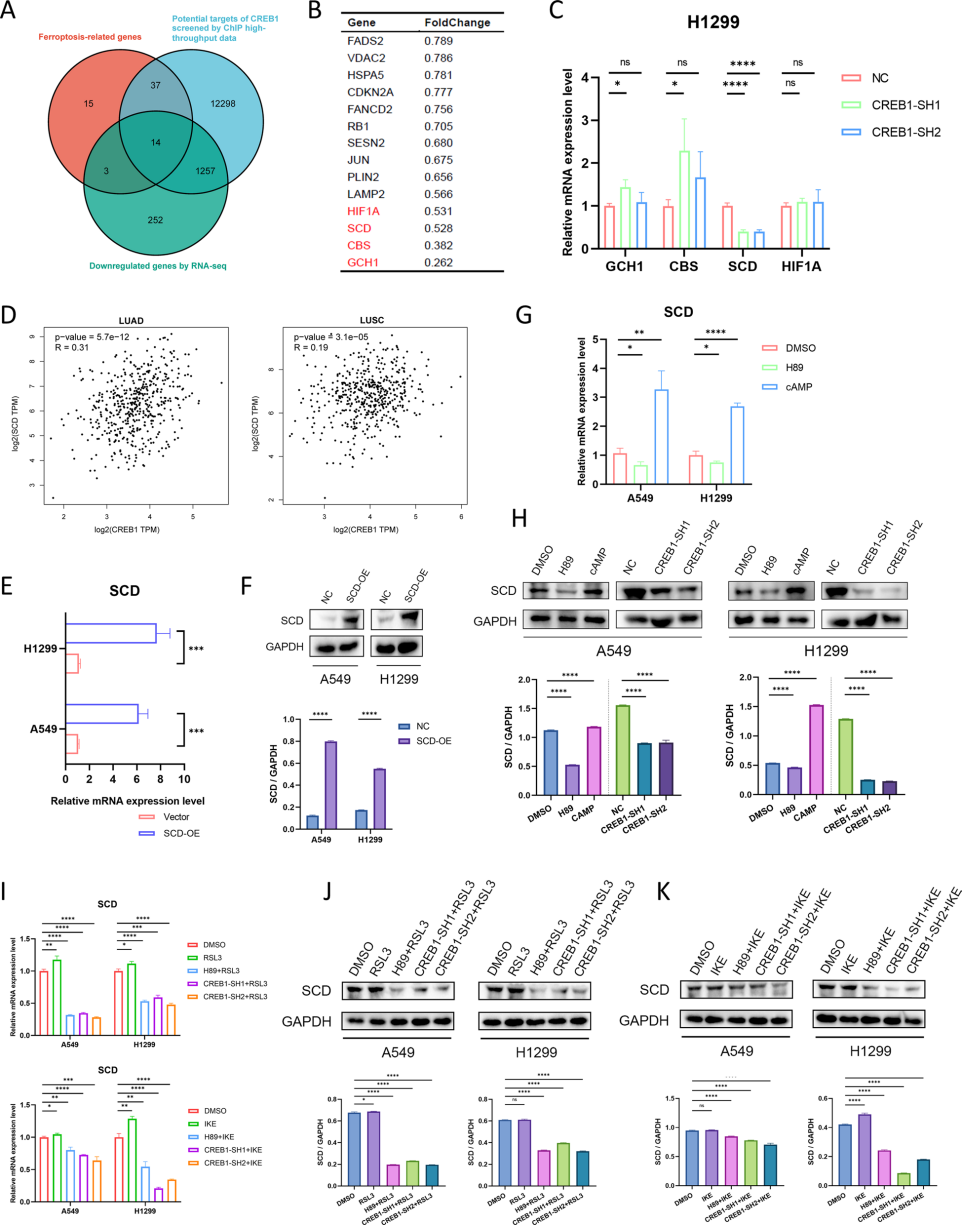
SCD is the downstream target of CREB1. **A** Selection of downstream genes of CREB1 by the intersection of ferroptosis-related genes, target genes of CREB1 screened by ChIP high-throughput data, and downregulated genes in CREB1-knockdown A549 cells revealed by RNA-seq. **B** Fifteen potential target genes of CREB1 with the most pronounced fold change in CREB1-knockdown A549 cells. **C** RT-qPCR revealed the mRNA expression of GCH1, CBS, SCD, and HIF1A between NC and CREB1-knockdown H1299 cells. **D** Pearson correlation between the SCD and CREB1 expression in NSCLC tissue from TCGA database.** E**, **F** RT-qPCR (E) and immunoblotting (F, cropped blots) revealed the SCD expression in NSCLC cells after transfected with vector or SCD-overexpression lentivirus. **G** RT-qPCR revealed the mRNA expression of SCD in A549 and H1299 cells after treatment with H89 (30 μM) or cAMP (100 μM) for 48 h. **H** SCD level in A549 and H1299 cells (cropped blots) after transfection with NC/ CREB1-SH1/ CREB1-SH2 lentivirus or treatment with H89 (30 μM)/ cAMP (100 μM) for 48 h. **I**, **J**, **K** RT-qPCR (**I**) and Immunoblotting (cropped blots, **J** & **K**) revealed the SCD expression with indicating treatments in A549 and H1299 cells. NSCLC, non-small cell lung cancer. Data were analyzed by two-way ANOVA or Student’s t-test and were presented by mean ± SD from three biological replicates. ns, not significant; *, p < 0.05; **, p < 0.01; ***, p < 0.001; ****, p < 0.0001

We performed RNA-seq analysis in NC and CREB1-knockdown A549 cells to identify the downstream target of CREB1 in ferroptosis regulation of NSCLC. We focused on the genes that were downregulated by CREB1 knockdown, as CREB1 mainly acts as a transcriptional activator. We filtered the genes with absolute expression greater than 1 and a fold change less than 0.8 after CREB1 knockdown. Next, we intersected these genes with the ferroptosis-related genes from the FerrDb database (http://www.zhounan.org/) [18] and the potential targets of CREB1 from GTRD ChIP-seq datasets (http://www.gtrd.bioumi.org) (Fig. 4A), and obtained 14 candidate genes (Fig. 4B). We validated the expression of these candidate genes by qRT-PCR and found that CBS, GCH1, and HIF1A showed inconsistent trends in CREB1-knockdown A549 and H1299 cells (Fig. 4C). Interestingly, SCD mRNA expression was decreased in both A549 and H1299 cells after CREB1 knockdown (Fig. 4C). Besides, the correlation analysis revealed a positive correlation between CREB1 and SCD in NSCLC tissues (Fig. 4D).

To verify the effect of SCD on NSCLC, we overexpressed SCD in A549 and H1299 cells and confirmed the overexpression effect by qRT-PCR (Fig. 4E) and immunoblotting (Fig. 4F) assays. The effect of SCD on ferroptosis in NSCLC cells was assessed by cell viability and lipid peroxidation assays. We found that SCD overexpression diminished the cytotoxicity of FINs and lipid peroxidation, while SCD inhibitor A939572 (10 μM) exerted the opposite effect (Figure S1). Next, we treated NSCLC cells with H89 (30 μM) or cAMP (100 μM) for 48 h and observed that SCD mRNA expression was significantly reduced or increased, respectively (Fig. 4G). The immunoblotting assays confirmed the same trend of SCD protein expression in response to CREB1-knockdown or H89/cAMP treatment (Fig. 4H). When treated with RSL3 (0.2 μM for A549, 0.05 μM for H1299) or IKE (2 μM) for 48 h, the SCD mRNA and protein level was slightly increased, which may act as a cellular defense for ferroptosis, while in H89 incubated (30 μM, 48 h) or CREB1-knockdown NSCLC cells, the SCD was significantly diminished (Fig. 4I, J, K). Taken together, SCD was confirmed as the potential target gene of CREB1 in regulating ferroptosis of NSCLC.

### SCD reverses the effect of CREB1 knockdown on ferroptosis

**Fig. 5:**
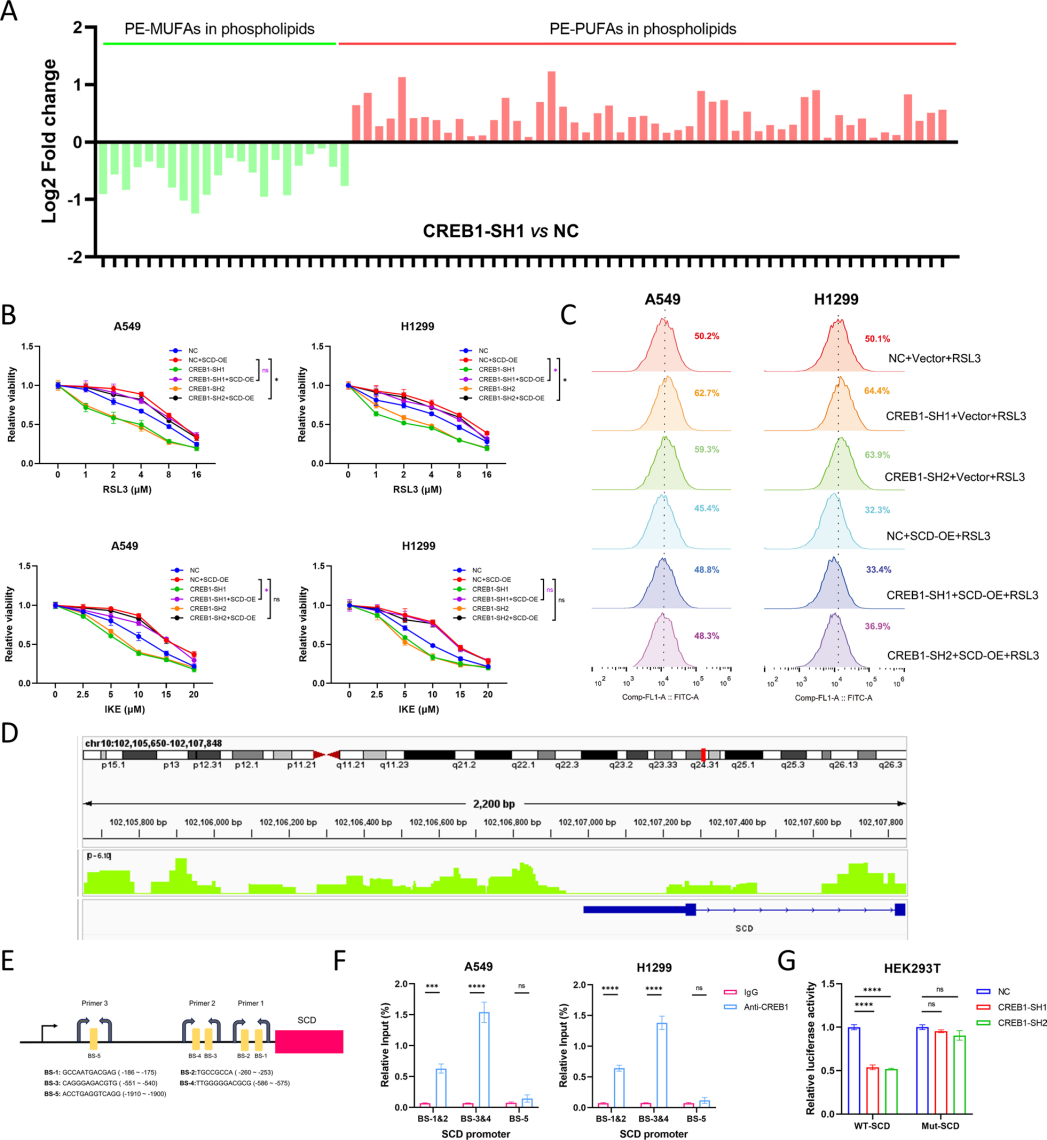
SCD reverses the effect of CREB1 knockdown on ferroptosis and CREB1 binds to the promoter region of SCD to promote SCD expression.** A** The histogram revealed the relative abundance of PE-PUFAs and PE-MUFAs in A549 cells transfected with NC or CREB1-SH1 lentivirus. **B**, **C** Cell toxicity assays (**B**) and lipid peroxidation measurement (**C**) demonstrated the rescue effect of SCD in CREB1-knockdown A549 and H1299 cells. **D** Predicted BS of CREB1 and design of primers for ChIP-qPCR. **E** ChIP-seq results demonstrated the CREB1-binding peak in the promoter region close to the TSS of SCD. **F** The enrichment of CREB1 in A549 and H1299 cells. **G** Dual-luciferase assays using luciferase plasmids containing WT or mutated type of the promoter region of SCD. PE, phosphatidylethanolamine; PUFAs, polyunsaturated fatty acids; MUFAs, monounsaturated fatty acids**;** BS, binding sites; ChIP, chromatin immunoprecipitation; TSS, transcription start site; WT, wild type. Data were analyzed by two-way ANOVA or Student’s t-test and presented by mean ± SD from three biological replicates. ns, not significant; *, p < 0.05; **, p < 0.01; ***, p < 0.001; ****, p < 0.0001

Since SCD is reported to participate in the biosynthesis of MUFAs to exert an anti-ferroptosis effect, we performed the pseudotargeted lipidomics analysis to investigate whether CREB1 is implicated in lipid metabolism. The histogram showed that the relative abundance of phosphatidylethanolamine-MUFAs (PE-MUFAs) was lower in CREB1-knockdown A549 cells, while PE-PUFAs available for lipid peroxidation demonstrated the opposite trend (Fig. 5A). At last, We found that SCD overexpression partially rescued the effect of CREB1 knockdown on ferroptosis and lipid peroxidation (Fig. 5B, C). These results indicate that SCD mediates the anti-ferroptotic function of CREB1 in NSCLC cells.

### CREB1 binds to the promoter region of SCD to promote SCD transcription

We performed ChIP-seq analysis to examine the binding of CREB1 to the promoter region of SCD (Fig. 5D) and found the significant enrichment of CREB1. Next, we used JASPAR (https://jaspar.genereg.net/) to identify the potential transcription factor binding sites (TFBS) of SCD and designed three primer pairs (Fig. 5E). We conducted ChIP assays and confirmed that CREB1 could bind to the BS-1&2 and BS-3&4 regions of the SCD promoter (Fig. 5F). Subsequently, the dual luciferase assay was conducted to verify whether CREB1 could directly activate SCD transcription. We constructed a firefly luciferase plasmid containing the promoter region of SCD and a mutant plasmid with all putative TFBS of SCD in this region randomly altered. The HEK-293 T cell was transfected with these plasmids after CREB1 knockdown. We observed that CREB1 knockdown significantly reduced the firefly luciferase activity in the cells transfected with the wild-type SCD promoter plasmid, but not in the cells transfected with the mutant SCD promoter one (Fig. 5G). These results indicate that CREB1 directly regulates SCD expression in NSCLC cells.

### CREB1 confers ferroptosis resistance in vivo

**Fig. 6:**
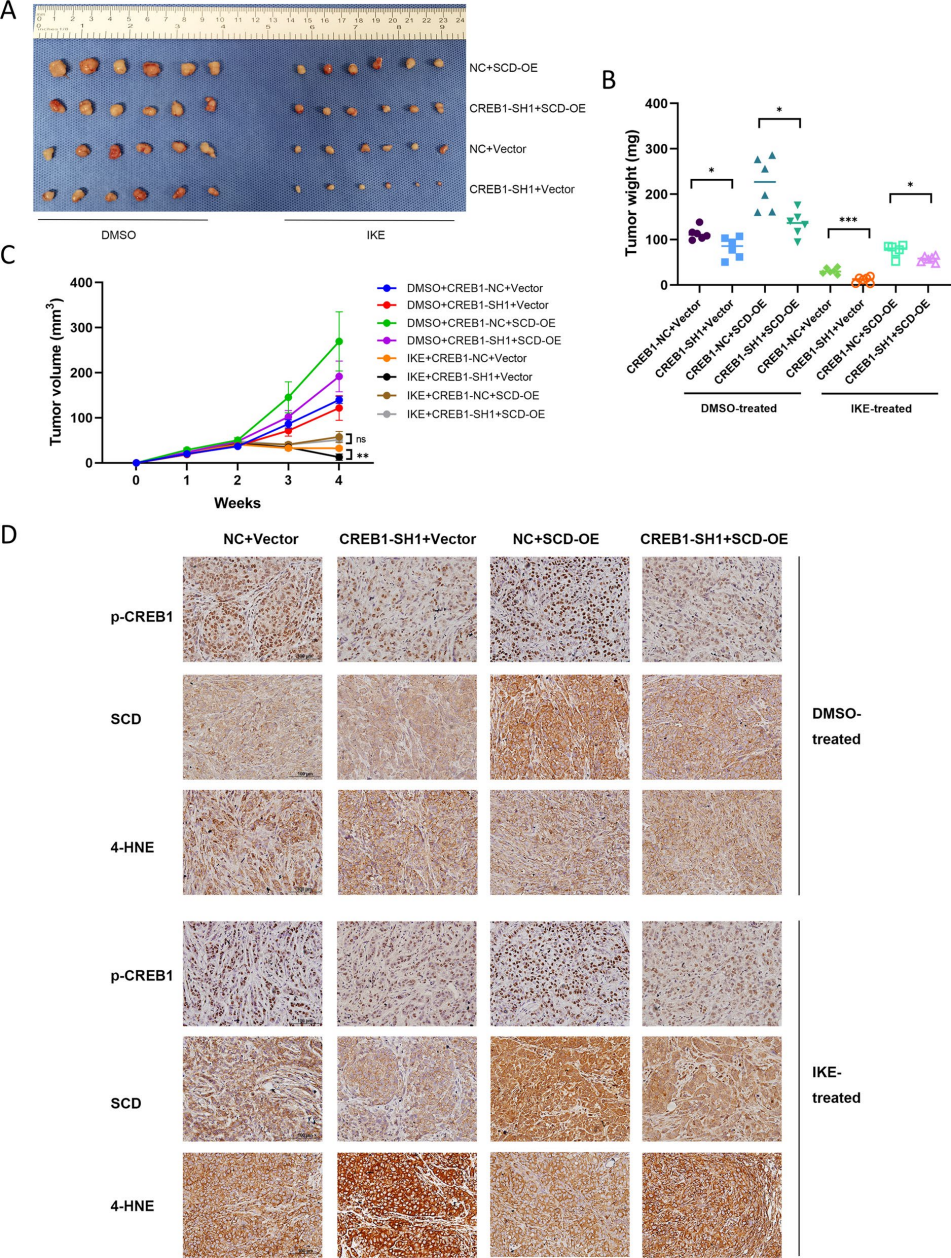
Knockdown of CREB1 confers sensitivity to IKE and could be rescued by SCD in vivo. **A** Tumors were resected after 4 weeks. **B** Tumor volumes were measured weekly. **C** The tumor weight of each group was determined after 4 weeks. **D** Representative images of immunohistochemistry staining of 4-HNE, p-CREB1, and SCD from different groups. 4-HNE, 4-Hydroxynonenal. Data were analyzed by two-way ANOVA and were presented by mean ± SD from six biological replicates. ns, not significant; *, p < 0.05; **, p < 0.01; ***, p < 0.001

To verify the result in vivo, we performed xenograft tumor formation assays in male nude mice. DMSO or IKE (30 mg/kg) was intraperitoneally injected every three days for six times. We found that IKE significantly inhibited tumor growth compared to the DMSO-treated group (Fig. 6A). In the IKE-treated group, CREB1 knockdown reduced the tumor volume and weight, and this effect was reversed by SCD overexpression (Fig. 6B, C). The IHC staining of the xenograft tumor tissues showed that CREB1 knockdown decreased the level of 4-HNE, a marker of lipid peroxidation, and that SCD overexpression restored the CREB1 knockdown effect (Fig. 6D). These results indicate that CREB1 knockdown enhances ferroptosis and suppresses tumor growth in vivo, and that SCD overexpression counteracts this effect.

### High CREB1 expression is associated with poor prognosis in NSCLC patients

**Fig. 7:**
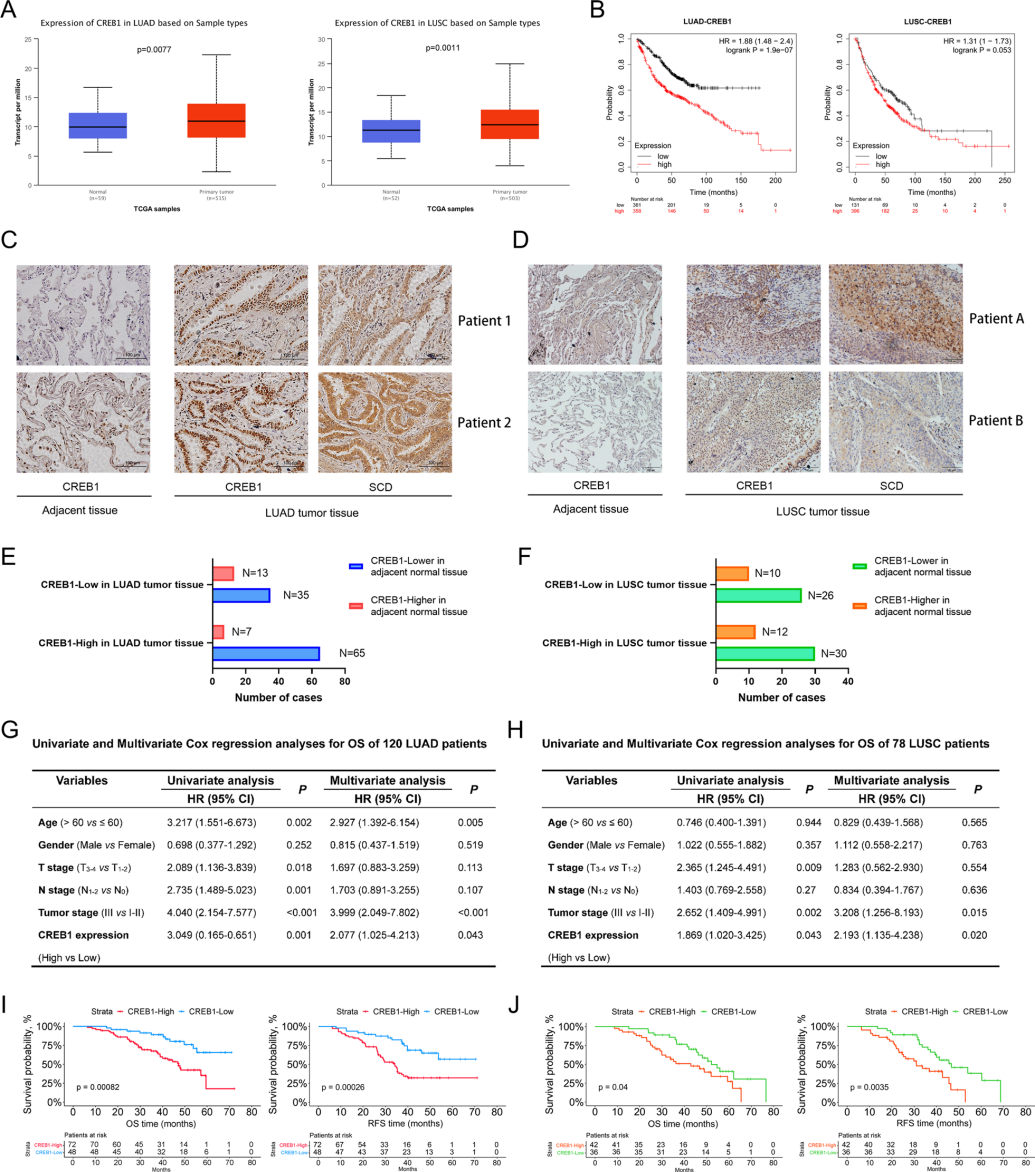
Clinical significance of CREB1 in NSCLC patients. **A** The mRNA expression of CREB1 in NSCLC tumor and adjacent normal tissue from TCGA database. **B** The Kaplan–Meier survival analysis of LUAD and LUSC patients divided by CREB1 expression. **C**, **D** Representative images of immunohistochemistry staining of 120 LUAD (**C**) and 78 LUSC (**D**) patient samples. **E**, **F** The numbers of LUAD (**E**) and LUSC (**F**) cases stratified by CREB1 expression. **G**, **H** Univariate and multivariate Cox regression analyses for OS of LUAD (**G**) and LUSC (**H**) patients. **I**, **J** Kaplan–Meier survival analysis with log-rank test demonstrated the OS and RFS in LUAD (**I**) and LUSC (**J**) patients with high or low CREB1 expression. OS, overall survival; RFS, recurrence-free survival

Compared with adjacent normal tissue, CREB1 is highly expressed in LUAD and LUSC tissue (Fig. 7A). Subsequently, we used the KM plotter web tool to perform survival analysis and found that high CREB1 expression was associated with poor OS in NSCLC patients (Fig. 7B). Next, 120 LUAD and 78 LUSC patients from our institution were divided into high and low groups according to the expression of CREB1, which is determined by IHC (Fig. 7C, D). Among the 120 LUAD patients, 72 had high CREB1 expression and 48 had low CREB1 expression (Fig. 7E). As for the 78 LUSC patients, 36 harbored high CREB1 expression and 42 harbored low CREB1 expression (Fig. 7F). Cox regression analysis revealed that CREB1 expression was an independent prognostic factor for LUAD and LUSC patients (Fig. 7G, H). Furthermore, Kaplan–Meier survival analysis indicated that high CREB1 expression correlated with shorter RFS and OS in our NSCLC cohorts (Fig. 7I, J).

## Discussion

PKA is a tetramer enzyme composed of two catalytic subunits and two regulatory subunits [14], which can be activated by cAMP and leads to the phosphorylation of the threonine and serine residues in substrate proteins. As the well-identified downstream target of PKA, activated CREB1 can bind to the cAMP response element in the promoter region of target genes to promote gene transcription, and then mediate a variety of biological processes [19]. Multiple studies have demonstrated the role of PKA/CREB1 in tumor biology, the effect of which varies with tumor types and even the same kind of malignancy. It is found that in H1299 cells, the PKA/CREB1 axis could favor cell proliferation by targeting RGS17 [20]. Similarly, the treatment of PKA inhibitor H89 mitigated hypoxia-induced epithelial-mesenchymal transition, cell migration, invasion, and of lung cancer cells and reduced tumor size in vivo [21, 22]. Another study found that the administration of MHY4571 induces apoptosis via inhibiting PKA/CREB1 pathway in LUSC [23]. For colorectal cancer, Teruaki et al. found that the cAMP/PKA/CREB1 axis plays an important role in maintaining the stemness and metastatic capability of cancer cells, suggesting that CREB1 may be a potential target for treating metastatic colorectal cancer [24]. On the contrary, the administration of forskolin (the agonist of adenylyl cyclases) was reported to sensitize lung cancer cells to radiotherapy-induced apoptosis via PKA-mediated PP2A phosphorylation [25]. Collectively, the effect of PKA/CREB1 pathway may be subject to the different contexts in lung cancer. Tocladesine, a cAMP analog, has been demonstrated to prevent the development of various malignancies in vitro and in vivo, including lung, breast, and leukemia [26]. Although the underlying mechanisms of tocladesine remain unknown, there have been published phase I and II clinical trials to investigate the effectiveness of tocladesine in metastatic colorectal cancer (NCT00021268) and multiple myeloma (NCT00004902).

Ferroptosis is an iron-dependent form of regulated cell death caused by excessive peroxidation of PUFAs and resultant membrane rupture [27]. Accumulating preclinical studies suggest that the induction of ferroptosis may be an effective method for the treatment of malignancies. However, the effect of PKA/CREB1 pathway in regulating ferroptosis of NSCLC still needs to be fully illuminated. In this study, we discovered that the NSCLC cells were more susceptible to ferroptosis after treatment with PKA inhibitor H89, while PKA agonist cAMP exerted the opposite effect. It is rational that the application of PKA/CREB1 inhibitors may potentiate the efficacy of ferroptosis inhibitors in treating NSCLC patients. Up to now, preclinical research has confirmed the tumor-suppressing properties of several PKA inhibitors, nevertheless, none of them were tested in human clinical trials [28]. The investigation of an efficacious and safe inhibitor of PKA/CREB1 axis is warranted.

Fatty acid metabolism is a key factor for the mediation of cell susceptibility to ferroptosis. Typically, fatty acids can be divided into SFAs, PUFAs, and MUFAs. In contrast to SFAs, which can be obtained by dietary consumption or de novo synthesis, PUFAs can only be obtained through diet due to the absence of corresponding desaturases in mammals [29]. PUFAs have bis-allylic hydrogen atoms that are easily removed by oxidation and thus are susceptible to lipid peroxidation, which is a key event in ferroptosis initiation [30]. SCD (also known as SCD1), which is positioned in endoplasmic reticulum, plays a crucial role in catalyzing the conversion from SFAs to MUFAs by introducing a single double bond in a spectrum of methylene-interrupted fatty acyl-CoA substrates [31]. As the preferred substrates of SCD, palmitoyl- and stearoyl-CoA are transformed into palmitoleoyl- and oleoyl-CoA, respectively [32]. It has been demonstrated that MUFAs can compete with PUFAs to incorporate into the plasma membrane, thus reducing the amount of lipids containing oxidizable PUFAs that are accessible for lipid peroxidation and subsequent ferroptosis [9].

Research has uncovered that the STK11/KEAP1 co-mutation in LUAD cells leads to resistance to pharmacologically induced ferroptosis, which is largely dependent on the marked upregulation of SCD expression [33]. In ovarian cancer, SCD expression or palmitoleic acid supplementation (a type of MUFA) can confer ferroptosis resistance to cancer cells [10]. In this study, we confirmed that SCD is the target of CREB1, and the pro-ferroptosis effect exerted by CREB1 knockdown could be reversed by the overexpression of SCD in vitro and in vivo, highlighting the significance of fatty acid metabolism in ferroptosis.

It is discovered that tumor cells which were refractory to conventional therapies or prone to metastasize still exhibit high vulnerability to ferroptosis [5], highlighting the effect of ferroptosis in the treatment of patients with malignancies. Therefore, the combination of FINs and conventional therapies may offer a new therapeutic option for patients with refractory malignancies. Some drugs (such as sulfasalazine, sorafenib, and statins) have been shown to have pro-ferroptotic effects in pre-clinical studies, among which statins may be a promising candidate for further clinical trials. However, the toxicity of pro-ferroptotic agents, such as bone marrow injury, should also be considered. In this study, we demonstrated that the inhibition of PKA/CREB1 pathway increased the susceptibility of NSCLC cells to ferroptosis, suggesting that targeting this pathway may be a novel strategy for the treatment of NSCLC patients.

## Conclusion

Our study reveals a previously unknown role of the PKA/CREB1/SCD axis in regulating ferroptosis in NSCLC. We demonstrate that inhibition of the PKA/CREB1 pathway sensitizes NSCLC cells to ferroptosis, which can be rescued by overexpression of SCD both in vitro and in vivo. These findings provide new insights into the molecular mechanisms of ferroptosis and suggest potential therapeutic targets for NSCLC.

### Supplementary Information

**Additional file 1:** Summary of the primers and primary antibodies used in this study.

**Additional file 2: Table S1.** Summary of clinical information of LUAD patients by groups of CREB1 expression. **Table S2.** Summary of clinical information of LUSC patients by groups of CREB1 expression.

**Additional file 3: Figure S1.** **A**, **B** Relative cell viablility of A549 and H1299 cells exposed to increasing concentrations of RSL3 after pretreatment with SCD inhibitor A9397572 (10 µM) for 24 h. **C**, **D** Relative cell viablility of NSCLC cells exposed to increasing concentration of RSL3 after transfection with vector or SCD-overexpression lentivirus. **E**, **F** Effect of SCD modulation on lipid peroxidation in A549 and H1299 cells. Cells were either transfected with SCD over-expression lentivirus or pretreated with A939572 (10 µM) for 24 h, and then treated with RSL3 (2 µM) for 2h.

## Data Availability

The inquiry of original data generated in this study can be directed to the corresponding authors for rational reasons.
