# Supplementary material for: Inhibition of PKA/CREB1 pathway confers sensitivity to ferroptosis in non-small cell lung cancer
Source: Respir Res. 2023 Nov 13;24:277. doi: 10.1186/s12931-023-02567-3 (PMC10644539; doi:10.1186/s12931-023-02567-3)
Supplement: Supplementary file 1 — Additional file 1: Summary of the primers and primary antibodies used in this study. [file 12931_2023_2567_MOESM1_ESM.docx]

**Additional file 1**

| **Primers for**  **RT-qPCR** | **Sequence** | **Source** |
| --- | --- | --- |
| ActinB | F: CTGGGACGACATGGAGAAAA  R: AAGGAAGGCTGGAAGAGTGC | Sangon Biotech |
| CREB1 | F: CACCGTTACAGTGGTGATGG  R: AAGGGCTACCAGGAGACCATCT | Sangon Biotech |
| SCD | F: TCTAGCTCCTATACCACCACCA  R: TCGTCTCCAACTTATCTCCTCC | Sangon Biotech |
| CBS | F: ACACCACCGCTGATGAGATCCT  R: CATCGTTGCTCTTGAACCACTTGTC | Sangon Biotech |
| GCH1 | F: AAGGGCTACCAGGAGACCATCT  R: CAAGGCTTCCGTGATTGCTACAG | Sangon Biotech |
| HIF1A | F: ACTGATGACCAGCAACTTGAGGAAG  R: CCATCGGAAGGACTAGGTGTCTGAT | Sangon Biotech |
| CBS | F: ACACCACCGCTGATGAGATCCT  R: CATCGTTGCTCTTGAACCACTTGTC | Sangon Biotech |

| **Primary antibodies** | **Producer** | **ID/ Number** | **Dilution** |
| --- | --- | --- | --- |
| ActinB | Beyotime | AF5001 | 1:2000 |
| GAPDH | Beyotime | AG019-1 | 1:2000 |
| CREB1 | Abways | CY5426 | 1:2000 |
| phospho-CREB1 (Ser133) | Abways | CY5043 | 1:2000 |
| SCD | Absin | abs14771 | 1:2000 |
| PKAα/β CAT | Abways | AY0344 | 1:2000 |
| phospho-PKAα/β CAT | Abways | AY0345 | 1:2000 |

| **Primers for**  **CHIP-qPCR** | **Sequence** | **Source** |
| --- | --- | --- |
| Primer 1 | F: TGAGAAGGAGAAACAGAGG  R: AATGCTAATGAGGCTTCTGT | Sangon Biotech |
| Primer 2 | F: CCACCACTAACATCTCCGT  R: CATTGTTCGCAGGCGTAC | Sangon Biotech |
| Primer 3 | F: GGGTTCAAGAAATTCTCCTG  R: GCATGTGGATCACCTGAG | Sangon Biotech |
