# Supplementary material for: Inhibition of PKA/CREB1 pathway confers sensitivity to ferroptosis in non-small cell lung cancer
Source: Respir Res. 2023 Nov 13;24:277. doi: 10.1186/s12931-023-02567-3 (PMC10644539; doi:10.1186/s12931-023-02567-3)
Supplement: Supplementary file 2 — Additional file 2: Table S1. Summary of clinical information of LUAD patients by groups of CREB1 expression. Table S2. Summary of clinical information of LUSC patients by groups of CREB1 expression. [file 12931_2023_2567_MOESM2_ESM.docx]

**Additional file 2: Table S1 Summary of clinical information of LUAD patients by groups of CREB1 expression**

|  | **High** | **Low** | **p-value** |
| --- | --- | --- | --- |
|  | ***N=72*** | ***N=48*** |  |
| **Age** |  |  | 0.209 |
| <60 | 25 (34.7%) | 23 (47.9%) |  |
| >60 | 47 (65.3%) | 25 (52.1%) |  |
| **Stage** |  |  | 0.234 |
| I | 31 (43.1%) | 27 (56.2%) |  |
| II | 22 (30.6%) | 14 (29.2%) |  |
| III | 19 (26.4%) | 7 (14.6%) |  |
| **Gender** |  |  | 0.970 |
| Female | 42 (58.3%) | 29 (60.4%) |  |
| Male | 30 (41.7%) | 19 (39.6%) |  |
| **T** |  |  | 0.231 |
| 1 | 37 (51.4%) | 30 (62.5%) |  |
| 2 | 15 (20.8%) | 12 (25.0%) |  |
| 3 | 16 (22.2%) | 4 (8.33%) |  |
| 4 | 4 (5.56%) | 2 (4.17%) |  |
| **N** |  |  | 0.235 |
| 0 | 36 (50.0%) | 31 (64.6%) |  |
| 1 | 22 (30.6%) | 12 (25.0%) |  |
| 2 | 14 (19.4%) | 5 (10.4%) |  |
| **SCD expression** |  |  | 0.019 |
| High | 44 (61.1%) | 18 (37.5%) |  |
| Low | 28 (38.9%) | 30 (62.5%) |  |

**Additional file 2: Table S2 Summary of clinical information of LUSC patients by groups of CREB1 expression**

|  | **High** | **Low** | **p-value** |
| --- | --- | --- | --- |
|  | ***N=42*** | ***N=36*** |  |
| **Age** |  |  | 0.455 |
| <60 | 13 (31.0%) | 15 (41.7%) |  |
| >60 | 29 (69.0%) | 21 (58.3%) |  |
| **Stage** |  |  | 0.864 |
| I | 15 (35.7%) | 15 (41.7%) |  |
| II | 14 (33.3%) | 11 (30.6%) |  |
| III | 13 (31.0%) | 10 (27.8%) |  |
| **Gender** |  |  | 0.295 |
| Female | 22 (52.4%) | 24 (66.7%) |  |
| Male | 20 (47.6%) | 12 (33.3%) |  |
| **T** |  |  | 0.896 |
| 1 | 22 (52.4%) | 21 (58.3%) |  |
| 2 | 9 (21.4%) | 8 (22.2%) |  |
| 3 | 8 (19.0%) | 6 (16.7%) |  |
| 4 | 3 (7.14%) | 1 (2.78%) |  |
| **N** |  |  | 0.088 |
| 0 | 21 (50.0%) | 19 (52.8%) |  |
| 1 | 16 (38.1%) | 7 (19.4%) |  |
| 2 | 5 (11.9%) | 10 (27.8%) |  |
| **SCD expression** |  |  | 0.037 |
| High | 25 (59.5%) | 12 (33.3%) |  |
| Low | 17 (40.5%) | 24 (66.7%) |  |
