# Supplementary figures and images for: Inhibition of PKA/CREB1 pathway confers sensitivity to ferroptosis in non-small cell lung cancer
Source: Respir Res. 2023 Nov 13;24:277. doi: 10.1186/s12931-023-02567-3 (PMC10644539; doi:10.1186/s12931-023-02567-3)

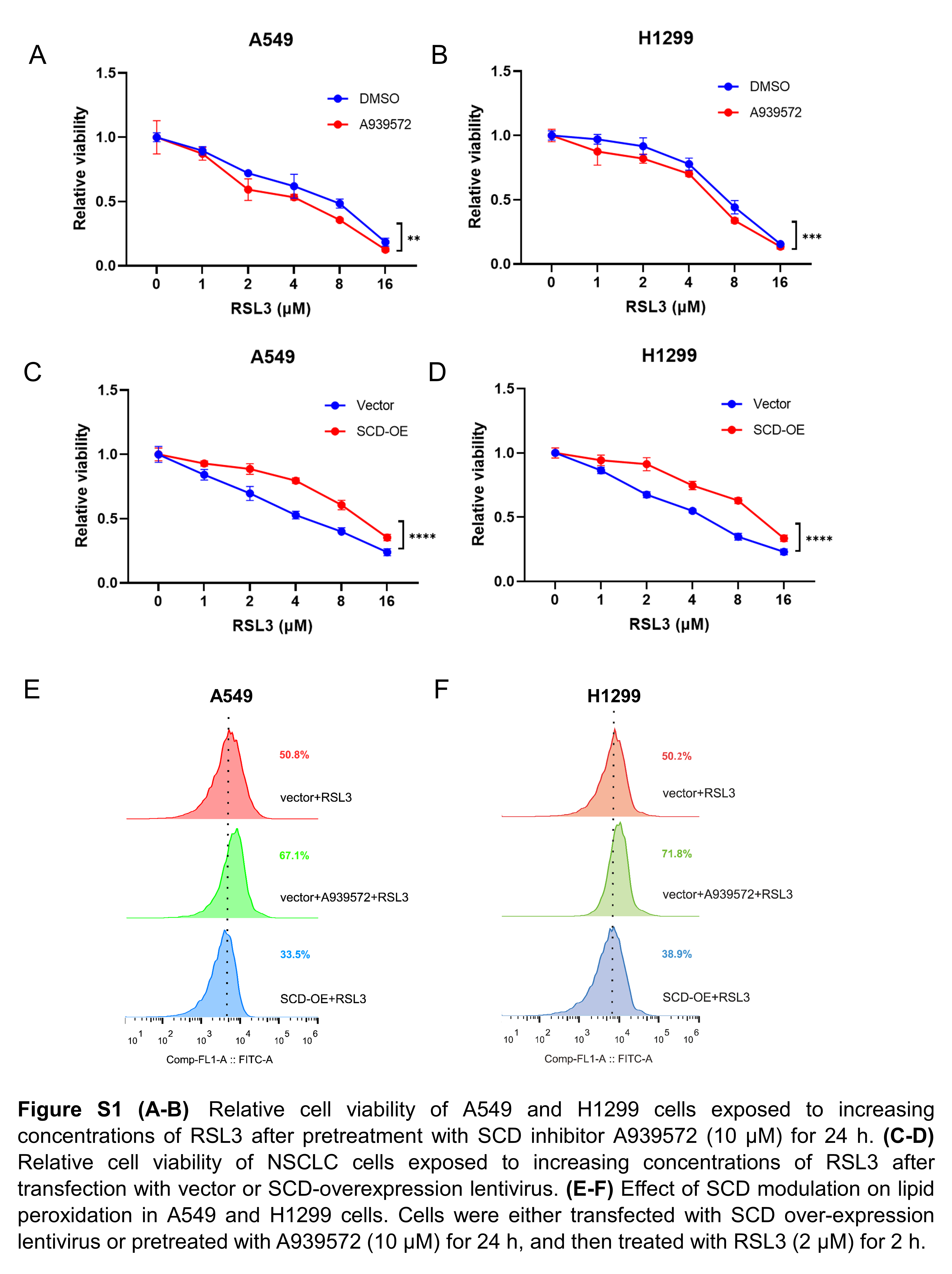

Supplement: Supplementary file 3 — Additional file 3: Figure S1. A, B Relative cell viablility of A549 and H1299 cells exposed to increasing concentrations of RSL3 after pretreatment with SCD inhibitor A9397572 (10 µM) for 24 h. C, D Relative cell viablility of NSCLC cells exposed to increasing concentration of RSL3 after transfection with vector or SCD-overexpression lentivirus. E, F Effect of SCD modulation on lipid peroxidation in A549 and H1299 cells. Cells were either transfected with SCD over-expression lentivirus or pretreated with A939572 (10 µM) for 24 h, and then treated with RSL3 (2 µM) for 2h. [file 12931_2023_2567_MOESM3_ESM.tif]
